# Supplementary material for: Genetic influences on brain and cognitive health and their interactions with cardiovascular conditions and depression
Source: Nat Commun. 2024 Jun 18;15:5207. doi: 10.1038/s41467-024-49430-7 (PMC11189393; doi:10.1038/s41467-024-49430-7)
Supplement: Supplementary file 1 — Supplementary Information [file 41467_2024_49430_MOESM1_ESM.pdf]

Supplementary Information: Genetic influences on brain and cognitive health and their interactions with cardiovascular conditions and depression

Peter Zhukovsky[1,2,3], Earvin S Tio[3,4], Gillian Coughlan[5], David A. Bennett[6], Yanling Wang[6], Timothy J Hohman[7,8], Diego A Pizzagalli[9], Benoit H Mulsant[1,2], Aristotle N Voineskos*[1,2], Daniel Felsky*[2,3,4,10,11]

*equal contribution

[1]Campbell Family Mental Health Research Institute, Centre for Addiction and Mental Health, Toronto, ON M5T 1R8, Canada
[2]Department of Psychiatry, Temerty Faculty of Medicine, University of Toronto, Toronto ON M5T 1R8, Canada
[3]Krembil Centre for Neuroinformatics, Centre for Addiction and Mental Health, Toronto, ON, Canada
[4]Institute of Medical Science, Temerty Faculty of Medicine, University of Toronto, Toronto, ON M5S 1A8, Canada
[5]Department of Neurology, Massachusetts General Hospital, Boston, MA 02129, USA
[6]Department of Neurological Sciences, RUSH Medical College, Chicago, Illinois 60612, USA
[7]Vanderbilt Memory & Alzheimer's Center, Vanderbilt University Medical Center, Nashville, TN 37232, USA
[8]Vanderbilt Genetics Institute, Vanderbilt University Medical Center, Nashville, TN 37232, USA
[9]Department of Psychiatry, Harvard Medical School and Center for Depression, Anxiety and Stress Research, McLean Hospital, Belmont, MA 02478, USA
[10]Dalla Lana School of Public Health, University of Toronto, Toronto, ON M5S 1A8, Canada
[11]Rotman Research Institute, Baycrest Hospital, Toronto, ON M6A 2E1, Canada

*Please address correspondence to Dr Daniel Felsky (daniel.felsky@camh.ca) and Dr Aristotle Voineskos (aristotle.voineskos@camh.ca)

Overview

1. GWAS re-analysis including a more expansive set of covariates
2. Cross-validation analysis of GxE interactions
3. Summary of multiple testing correction approach
4. Postmortem brain gene expression analysis
5. UKB sample description
6. eQTL analyses in FUMA
7. Partial least squares regression analysis of a subregion of chromosome 17
8. Notable GxE SNPs of interest

Supplementary Section 1. GWAS re-analysis including a more expansive set of covariates

To test the impact of including different sets of covariates, we re-ran a selection of four GWAS including age x sex, age^2, as covariates in addition to age, sex, site and total intracranial volume as included in the main analysis and 40 genetic principal components instead of the 10 PCs included in the primary analysis[1–3]. The GWAS outcomes we selected were global cortical thickness and thickness of inferior frontal gyrus pars orbitalis, inferior frontal gyrus pars triangularis, and the supramarginal gyrus, given the prominence of these regions identified by our GxE interaction testing. We assessed 1) the impact of the expanded covariate set on risk loci mapped by FUMA and 2) the impact of the expanded covariate set on the wider distribution of effect sizes (Z-statistics from the meta-analysis of GWAS) and p-values for each cortical thickness phenotype.

In Supplementary Data 20 we show the summary statistics for the genomic risk loci in the main analyses and in the sensitivity analyses alongside each other. Among the 49 genomic risk loci from the four GWAS, we found that 7 did not replicate in the expanded covariate analysis, whereby replication required us to find a SNP from the genomic risk locus with LD $r^2>0.6$ with the SNP from the genomic risk locus from the main analyses. Further, we found three new variants using the expanded set of covariates.

Second, we show the impact of expanding the set of covariates on Z-statistics and p-values for these four GWAS in the Supplementary Figures 7 and 8, respectively. The correlations between the Z-statistics (including Z-statistics with uncorrected $p<0.05$) from the main analysis and from the analyses including an expanded set of covariates was very high ($r>0.999$, Supplementary Figure 7). Further, Bland-Altman plots[1] showed that the inclusion of an expanded set of covariates had an appreciable, but comparatively small impact on the significance of findings in the four GWAS (Supplementary Figure 8).

Supplementary Section 2. Cross-validation analysis of GxE interactions

In order to test the robustness of our GxE findings, we repeated all analyses using cross-validation. For each of the 220 candidate SNPs, we first split our sample in 10 folds. For each split, we fit linear models in 90% of the sample, creating a GxE interaction brain map (featuring 360 regions) for depression and cardiovascular disease. We then thresholded each brain map at uncorrected $p<0.01$ in the inner fold (90% of the sample), and proceeded to test for interactions in the outer fold, i.e. the held-out 10% of the data. For regions that passed the $p<0.01$ threshold in the inner fold, we averaged the cortical thickness values in the outer fold and obtained an interaction p-value. Where no significant regions were identified, we used global cortical thickness as an outcome. We repeated the process 10 times, testing each combination of held-out outer fold and inner fold data. This cross-validation procedure allowed us to test the robustness of the regions with an interaction in held-out data. As a result, for each SNP, we obtained a p-value for each of the cross-validation splits. We combined each set of 10 p-values across the splits using Fisher's method[4]. An overview of the approach is shown in Supplementary Figure 3.

The summary statistics for whole-sample analyses and cross-validation analyses for cardiovascular disease and depression are shown in Supplementary Data 21 and 22, respectively. We found that many of the SNPs that passed the stringent significance threshold in the main analyses ($p<0.05/220/18.1$ effective comparisons) also passed the uncorrected $p<0.05$ threshold in the cross-validation analyses. We found that 20 of the 60 SNPs with a significant interaction with cardiovascular disease in the main analyses were also significant at uncorrected $p<0.05$ following cross-validation. Among 46 SNPs with a significant interaction with depression in the main analyses, 7 SNPs were significant at uncorrected $p<0.05$ following cross-validation.

Supplementary Section 3. Summary of multiple testing correction approach

We conducted several sets of analyses with different significance thresholds chosen for the purpose of the analysis. In our GWAS studies, we used the conventional discovery threshold of $5x10^{-8}$. In Supplementary Data 3 and 4 we also indicate which variants pass the more stringent $5x10^{-8}/34=1.5x10^{-9}$ threshold, which accounts for 34 individual region cortical thickness GWAS using the Bonferroni method.

Next, in extension of our UKB GWAS findings, we analyzed the much smaller ROS/MAP sample. In the first set of analyses of ROS/MAP genotype data (n=202), we tested for associations between the 220 SNPs identified as genomic risk loci for cortical thickness in UKB analyses and cortical thickness in ROS/MAP. When testing whether loci associated with cortical thickness *of a specific region* in our GWAS were associated with *the thickness of the same region* in ROS/MAP, we adopted a liberal pseudo-replication threshold of $p<0.05$. When testing whether genomic risk loci for a specific cortical thickness trait from the UKB were associated with any cortical thickness *in any region* in ROS/MAP, we adopted a more stringent threshold of 0.05/33, again using the Bonferroni method, since the space of tests was now expanded 33 times for each GWAS-implicated gene. In the second set of analyses of the ROS/MAP RNASeq data (n=66), we have tested for differential gene expression associated with cortical thickness of the rostral middle frontal and caudal middle frontal regions. We aimed to identify genes that were associated with cortical thickness both in the ROS/MAP differential gene expression analyses at a liberal pseudo-replication threshold of $p<0.05$ and in the UKB eQTL analyses.

Next, we tested for the associations between cognitive function, measured in both CLSA and UKB, and the 220 unique SNPs representing genomic risk loci identified in our GWAS. We applied false discovery rate (FDR) correction $p_{FDR}<0.1$ to each cognitive outcome. Each cognitive outcome (memory in CLSA, executive function in CLSA, paired associated learning in UKB, and fluid intelligence in UKB) was treated as a separate analysis, with multiple comparison correction applied within each analysis. We also generated 34 polygenic risk scores in CLSA using the summary statistics from our UKB GWAS, as described in the Methods. We tested for associations between memory and executive function in CLSA and these polygenic scores, reporting significant results at $p_{FDR}<0.1$ and suggestive effects at uncorrected $p<0.05$. We used a threshold of $p_{FDR}<0.1$ in the analyses of cognitive function for two reasons. First, our secondary analyses of cognitive function aimed to further explore and follow up on the GWAS findings in UKB; therefore, we aimed to have the most power to detect genetic effects on cognition. Second, previous studies of cognitive function of comparable sample size in UKB have identified only few independent SNP signals after LD clumping for verbal-numeric reasoning[5] and much larger samples (n>300,000) were needed to identify a large number of SNPs linked to general cognitive function[6]. Since our CLSA sample included just 25,387 participants, we acknowledge this as a limitation and have applied the threshold of $p_{FDR}<0.1$. CLSA participants are very healthy older adults, with very few participants with cognitive impairment[7,8], reducing the variability in cognitive performance.

Finally, we conducted a series of targeted GxE analyses. In our main GxE analyses in UKB, we corrected for the number of SNPs tested (220) and the number of effective cortical thickness comparisons (18.1), resulting in a conservative threshold of $p<1.3x10^{-5}$ ($p<0.05/220/18.1$). We then conducted a cross-validation analysis whereby the cortical thickness regions that we included in the interaction testing were generated in a separate split of the UKB data. We report the cross-validation effects at uncorrected $p<0.05$. When generating the mean regional cortical thickness value from the interaction brain maps (Supplementary Figure 3), we used a threshold of $p<0.01$ to ensure a substantial number of regions are included in testing the interactions for each of the 220 candidate SNPs. In the GxE analyses of polygenic risk scores, we tested for GxE interactions between cortical thickness polygenic scores for the 33 regions and executive function and memory in CLSA, reporting significant results at $p_{FDR}<0.1$ and suggestive effects at uncorrected $p<0.05$ following the rationale above. A limitation of our results is that our interaction findings are significant at $p_{FDR}<0.1$ but not at $p_{FDR}<0.05$.

Supplementary Section 4. Postmortem brain gene expression analysis

We provide the full summary statistics for the differential gene expression analyses in ROS/MAP in Supplementary Data 24 and Supplementary Data 25 for caudal middle frontal and rostral middle frontal thickness, respectively.

Supplementary Section 5. UKB sample description

In the UKB, we have conducted GWAS on participants of European ancestry, defined by genetic data from the Pan-UKBB consortium (https://pan.ukbb.broadinstitute.org/downloads/index.html). Extending our findings to non-European populations such as those included in the large, representative AllOfUs study (https://allofus.nih.gov/) will be critical in the future. We split the European sample into White British and Non-White British participants based on previous studies of the UKB data[9]. We used the definitions provided by Tanigawa et al[9], who state the following criteria: first, participants self-reported as having white British ancestry; second, their data was used to compute genetic principal components; third, they were not marked as outliers for heterozygosity and missing rates; fourth they did not show putative sex chromosome aneuploidy and finally, they had at most 10 putative third-degree relatives. While criteria 2-5 were related to genetic QC, the defining variable was self-reported ancestry. Running separate GWAS for the different European ancestries and meta-analyzing the results is a strength of our analyses, since it allows for separate effect estimation in different ancestries and thus for more model flexibility.

Supplementary Section 6. eQTL analyses in FUMA

We used FUMA's eQTL mapping package with default settings to map SNPs identified in select GWAS to genes[10]. FUMA maps SNPs to genes based on a significant eQTL association in GTEx (v8) brain cortex tissue and GTEx Frontal Cortex BA9 tissue data. We selected these tissue types given that the phenotypes of interest were cortical regions, and we included BA9 specifically given its overlap with the ROS/MAP postmortem tissue. Significant SNP-gene pairs were defined using false discovery rate-corrected $p<0.05$. The eQTL analysis thus allows us to identify genes, whose expression was significantly associated with allelic variation at the SNP in GTEx v8.

Supplementary Section 7. Partial least squares regression analysis of a subregion of chromosome 17

We used a partial least squares regression with 12 genetic risk loci SNPs from a subregion of chromosome 17 visualized in Figure 3 as predictors (34,660x12) and 13 cortical thickness variables as outcomes (34,660x13). We chose to apply this multivariate latent model instead of a more traditional

haplotype analysis given that a) haplotype construction from our discovered SNPs within such a large region with complex patterns of LD, including several unlinked variants, would have yielded many haplotypes with very low frequencies and b) we wanted to assess the relationships between all variants and several imaging phenotypes simultaneously. Both the SNPs and the cortical regions were selected based on the GWAS results shown in Supplementary Data 3. We regressed out age, age x sex, age^2, sex and TIV from the cortical thickness data and age, age x sex, age^2, sex, and the first 10 genetic PCs from the SNP data before entering the residuals into the PLS regression. We found that the overall PLS with 12 latent components model explained significantly more variance in cortical thickness than expected by chance[11,12] (Supplementary Figure 10A). We show the SNP loadings for the 12 latent variables in Supplementary Figure 10B and the correlations between latent variables and the cortical thickness data in Supplementary Figure 10C. While the first few latent variables captured more general patterns of association featuring multiple regions and genetic variants, we also found several variants with more specific associations with regional cortical thickness.

Supplementary Section 8. Notable GxE SNPs of interest

We found some SNPs that showed a significant interaction with cardiovascular conditions on cortical thickness in the UKB to also contribute to the polygenic scores that showed an interaction with cardiovascular conditions on executive function in CLSA. In particular, rs77690628 and rs3200031 have been associated with cortical thickness of the pars triangularis and pars orbitalis in our analyses and in previous studies [13] and also showed a significant interaction with cardiovascular conditions on cortical thickness. These SNPs have been mapped to the PP2R2A gene, which plays a role in negative control of cell growth and division (https://www.genecards.org/cgi-bin/carddisp.pl?gene=PPP2R2A).

Supplementary Figure 1. eQTL mapping of the complex locus influencing cortical thickness on Chromosome 17.

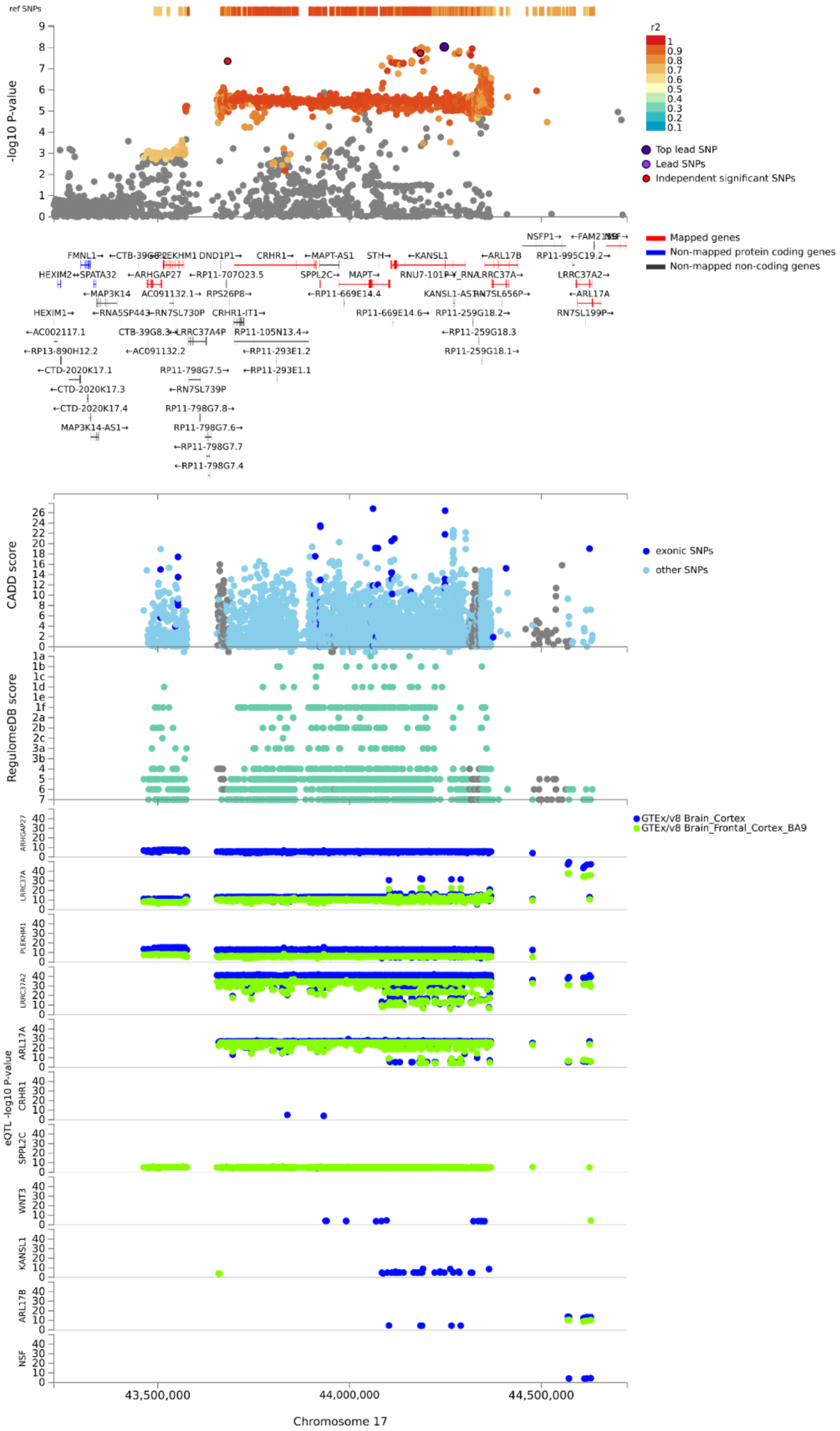

Supplementary Figure 2. A Seattle Alzheimer's Disease Brain Cell Atlas map of tissue-specific expression values for the *KANSL1*, *ARL17B*, *LRRC37A,* and *LRRC37B* genes, showing increased expression in microglia, endothelial cells, astrocytes, and oligodendrocytes. OPC: oligodendrocyte precursor cells; VLMC vascular and leptomeningeal cells

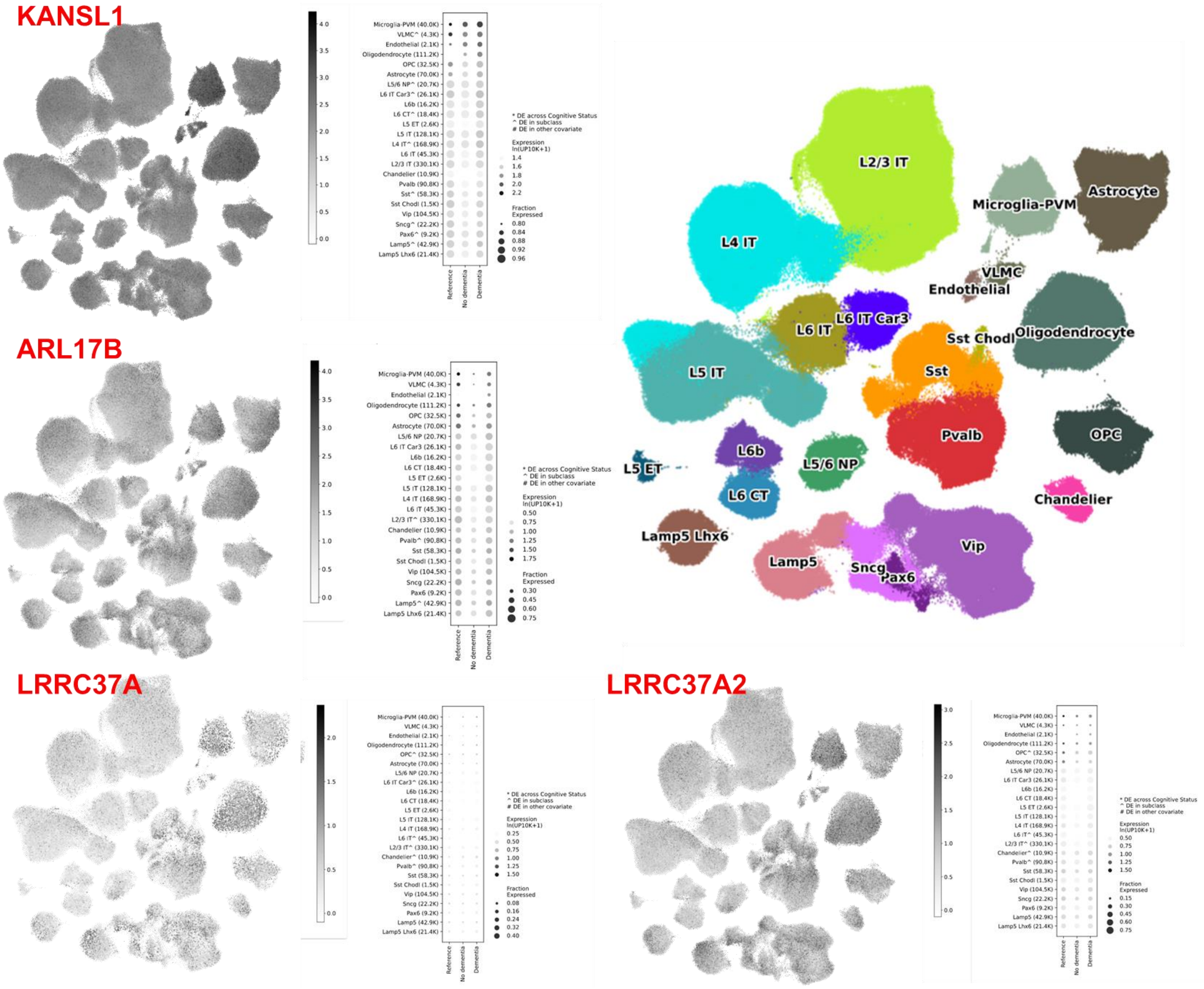

Supplementary Figure 3. Gene-by-environment interaction testing workflow and results.

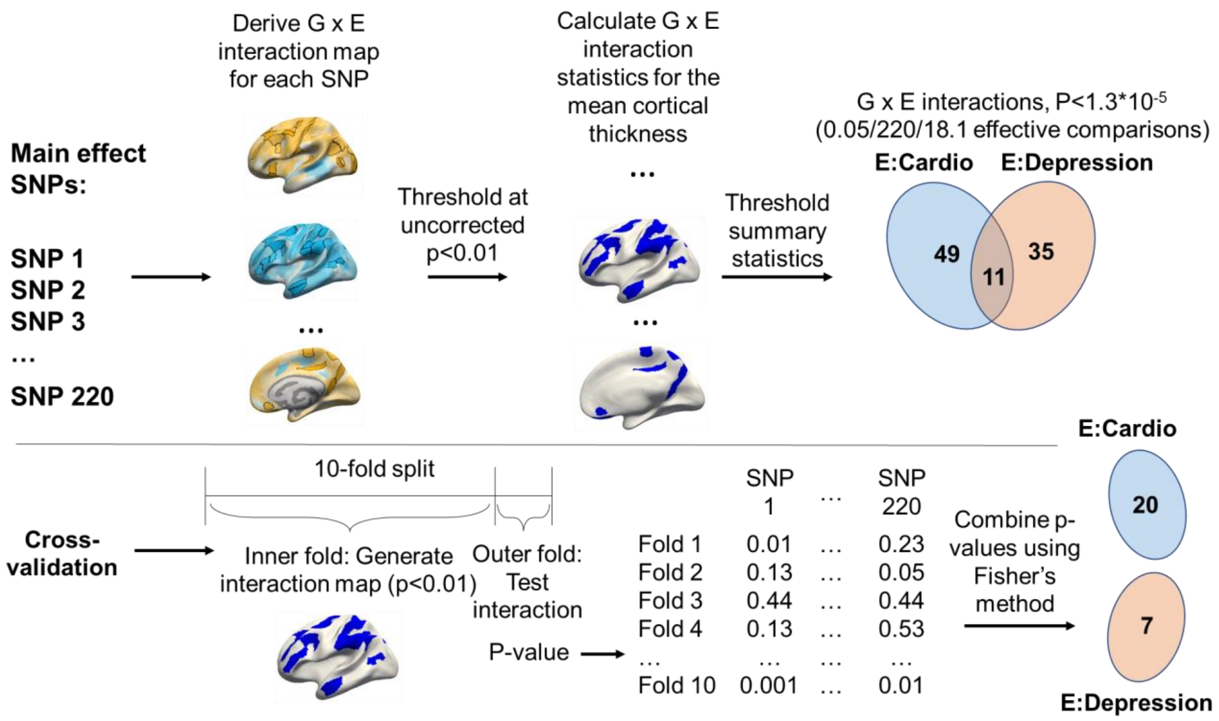

Supplementary Figure 4. Overview of interaction types, showing T-statistics for the main effect of SNPs on regional cortical thickness.

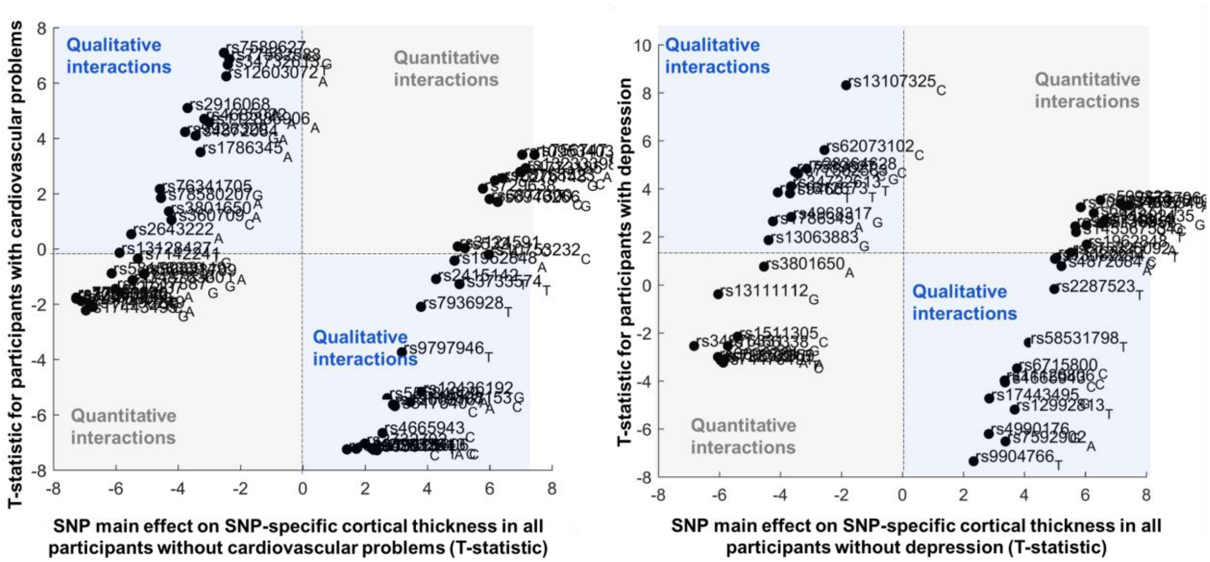

Supplementary Figure 5. Gene ontology analysis of GWAS findings. Genes obtained from positional mapping (FUMA ANNOVAR, Supplementary Data 10) were used in this analysis.

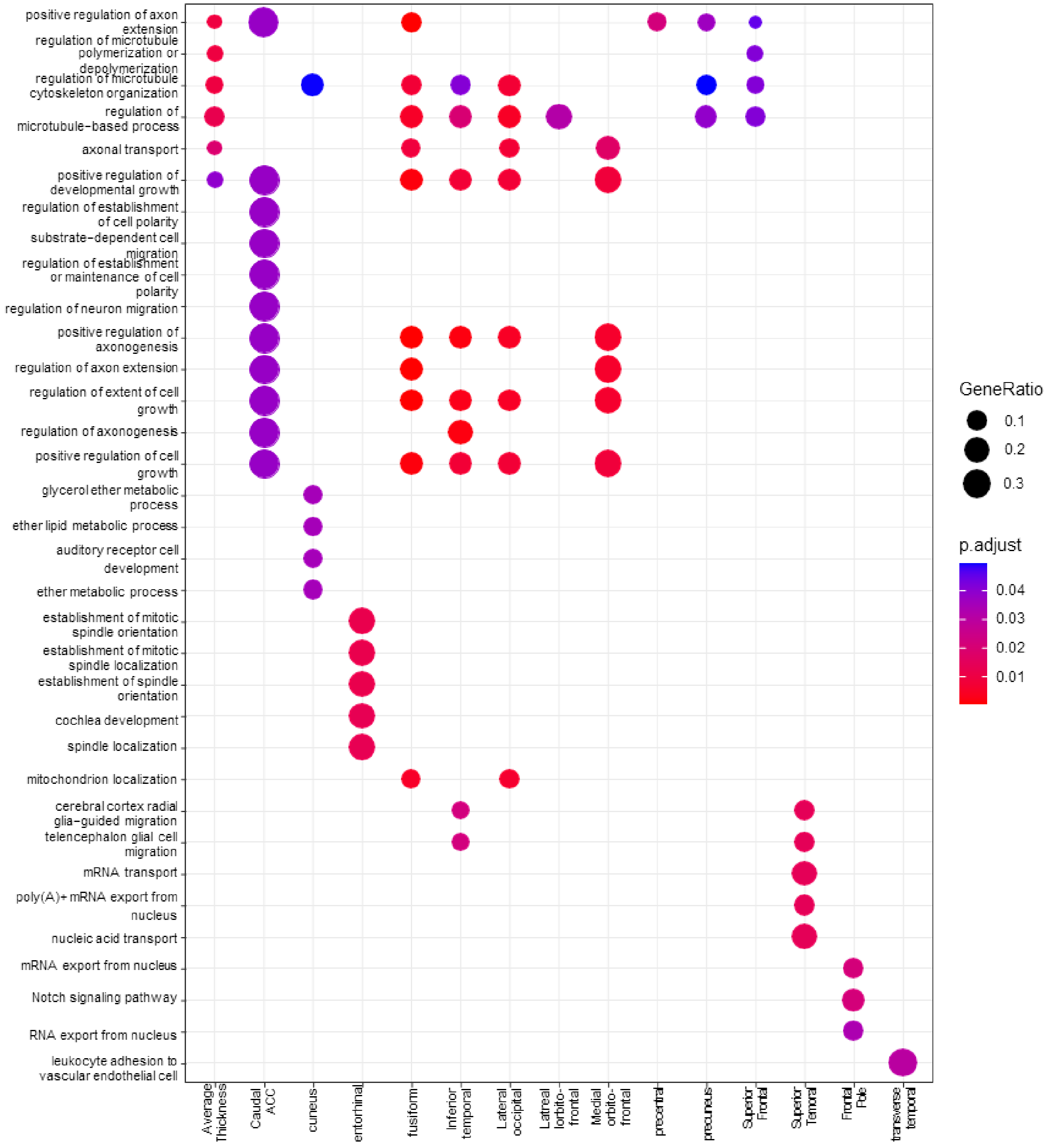

Supplementary Figure 6. Linkage disequilibrium plot between risk loci associated with cortical thickness in 17q21 (specifically the 43,009,797-44,353,728 region) and haplotype-tagging SNPs (htSNPs) for the *MAPT* gene. Associations with global cortical thickness are shown in red ($p<5x10^{-8}$), orange ($p<5x10^{-5}$) and gray ($p>0.001$) to denote significance. LD values shown are r^2, multiplied by 100.

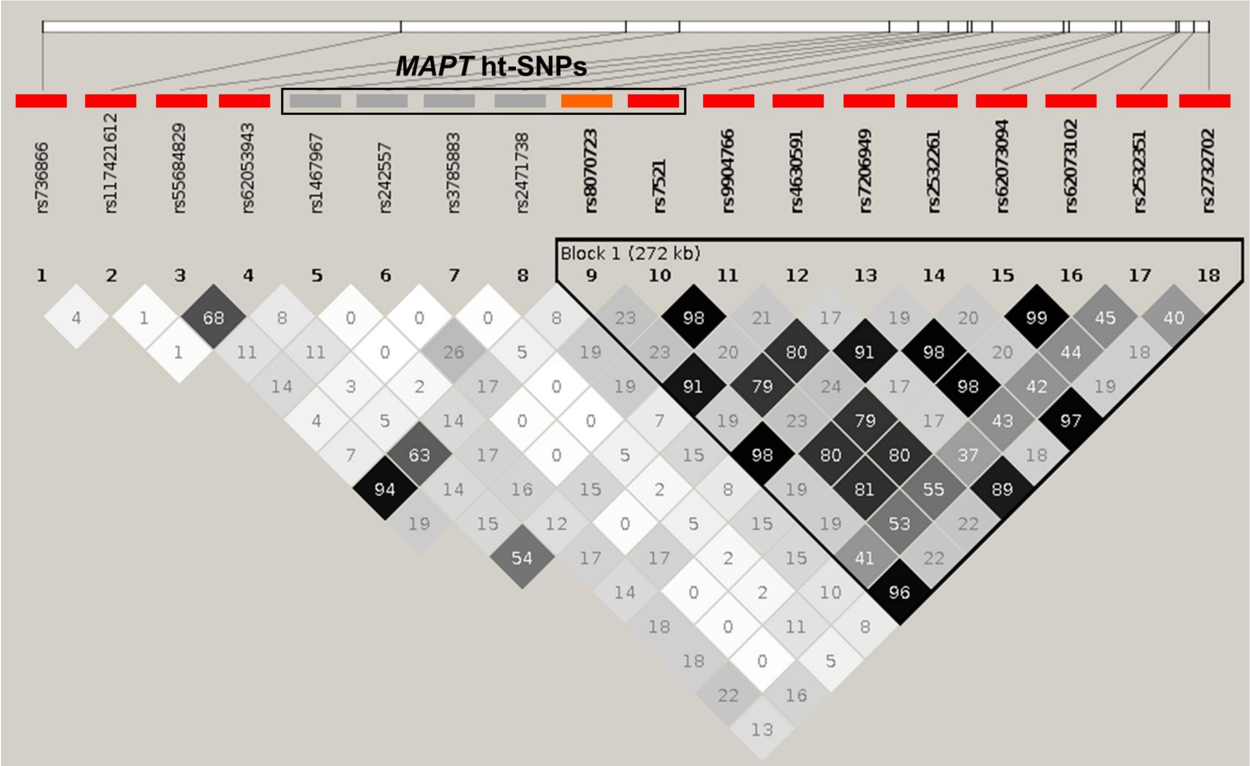

Supplementary Figure 7. Correlations between Z-statistics (derived from METAL) for all SNPs showing an association at p<0.05 with cortical thickness in main analyses (y axis) and in the supplementary analyses including an extended set of covariates for each of the four GWAS.

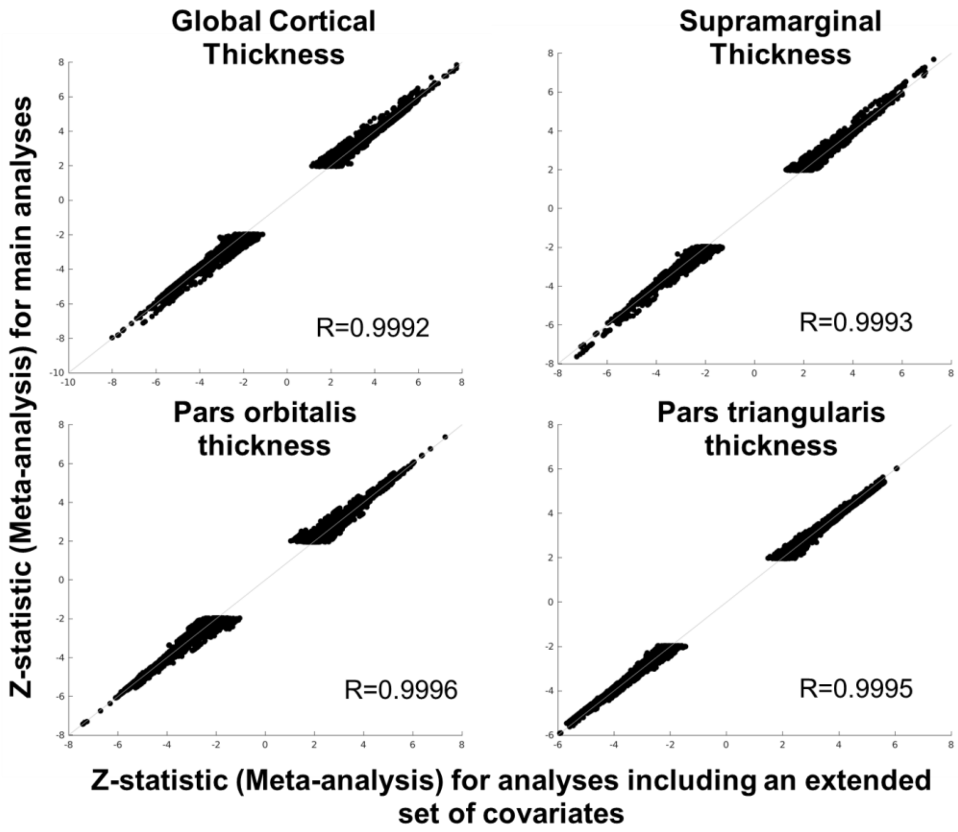

Supplementary Figure 8. Bland-Altman (BA) plots showing how associations between SNPs and cortical thickness in the four selected GWAS are affected by the addition of an extended covariate set. If the addition of extra covariates does not strongly affect the associations, the points should follow a horizontal distribution around y=0. On the other hand, if there are substantial differences in the significance of SNP-cortical thickness associations between the standard and extended set of covariates, we would see a shift away from the y=0 line. A strong downward shift would suggest reduced significance and potentially loss of genomic risk loci, while a strong upward shift would suggest the emergence of new loci that were not significant with the standard set of covariates. The plots show that the change in -log10 of P-values was relatively small, with the highest shift of -1.5 for global thickness and supramarginal thickness. A shift of 1 would correspond to a 10-fold change in p-value, for instance from 0.01 to 0.001.

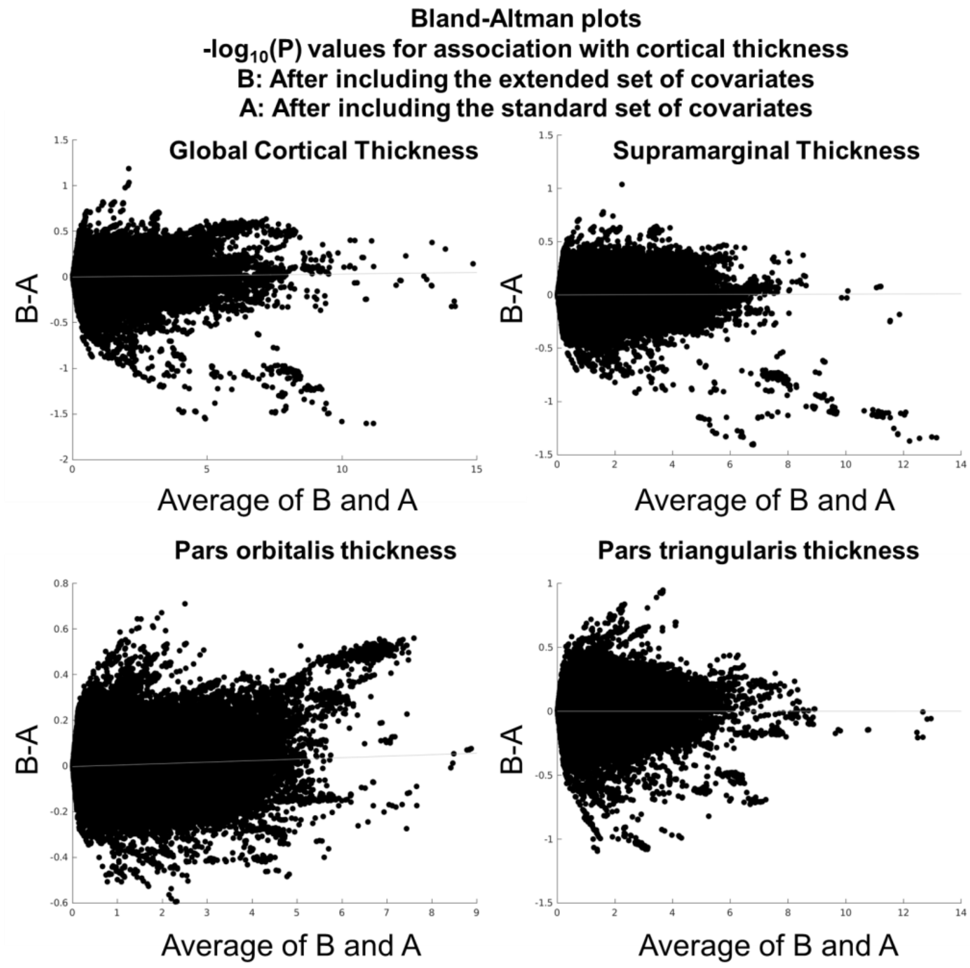

Supplementary Figure 9. Example interactions for rs11126806 (A), rs9926320 (B), and rs7575796 (C), showing the effect of genotype category stratified by the presence of cardiovascular conditions or depression (n=34,204 participants).

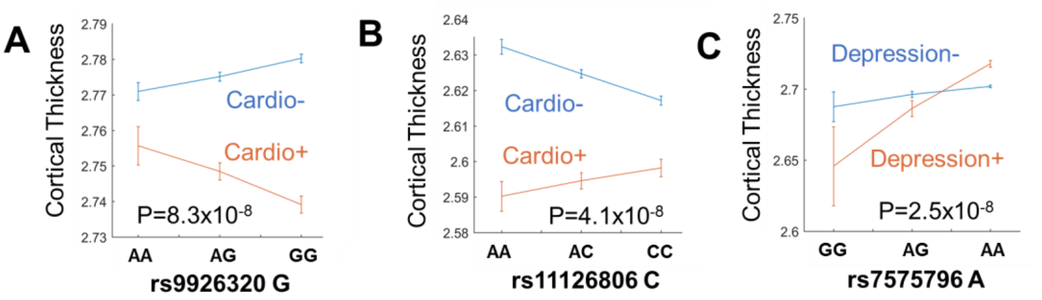

Supplementary Figure 10. Multivariate partial least squares (PLS) regression of genetic risk loci on chromosome 17 (located between 43,009,797 and 44,353,728) and cortical thickness phenotypes (n=34,204 participants). The PLS model explained significantly more variance in cortical thickness than expected by chance (A), with predicted vs observed correlations for the 13 cortical thickness phenotypes ranging between 0.038 and 0.059 (e.g. Superior frontal gyrus shown in panel D). Panel B shows the PLS loadings linking individual SNPs to the latent variables (LVs), with higher loadings indicating a greater contribution to the LVs. Panel C shows the correlations between latent variable scores and each cortical thickness (*p_{FDR}<0.1). arb. units: arbitrary units.

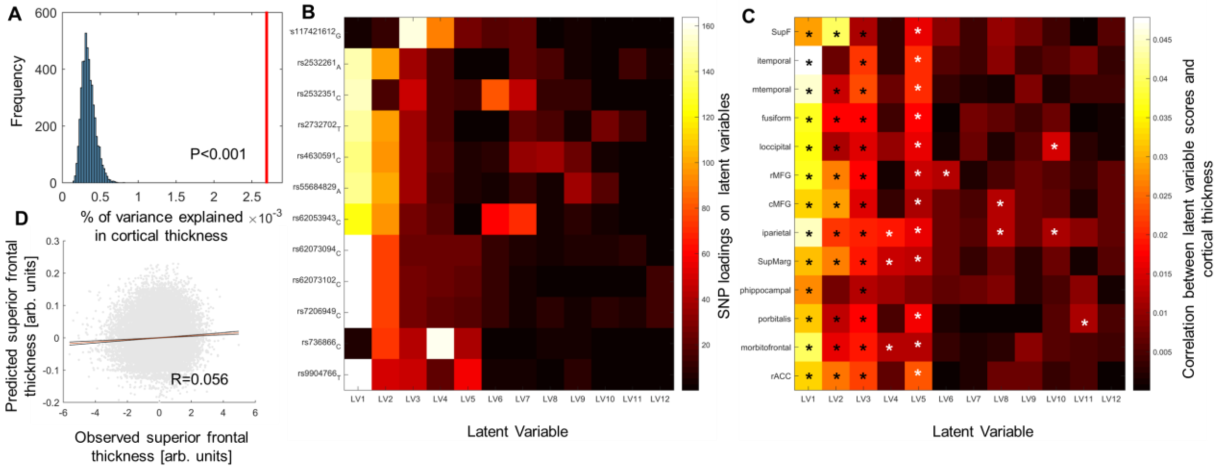

Supplementary Figure 11. Total intracranial volume (TIV) at baseline vs follow-up shows a very high degree of consistency (r=0.986, n=4,408 with baseline and follow-up data).

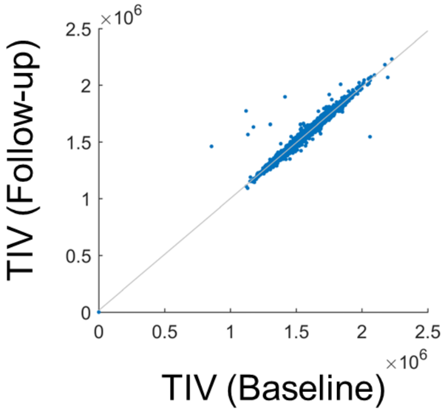

References:

1. Alfaro-Almagro, F. *et al.* Confound modelling in UK Biobank brain imaging. *Neuroimage* **224**, 117002 (2021).
2. Zhao, B. *et al.* Genome-wide association analysis of 19,629 individuals identifies variants influencing regional brain volumes and refines their genetic co-architecture with cognitive and mental health traits. *Nat. Genet.* **51**, 1637–1644 (2019).
3. Smith, S. M. *et al.* An expanded set of genome-wide association studies of brain imaging phenotypes in UK Biobank. *Nat. Neurosci.* **24**, 737–745 (2021).
4. Morgan, S. E. *et al.* Cortical patterning of abnormal morphometric similarity in psychosis is associated with brain expression of schizophrenia-related genes. *Proc. Natl. Acad. Sci.* 201820754 (2019) doi:10.1073/pnas.1820754116.
5. Davies, G. *et al.* Genome-wide association study of cognitive functions and educational attainment in UK Biobank (N=112 151). *Mol. Psychiatry* **21**, 758–767 (2016).
6. Davies, G. *et al.* Study of 300,486 individuals identifies 148 independent genetic loci influencing general cognitive function. *Nat. Commun.* **9**, 1–16 (2018).
7. Forgetta, V. *et al.* Cohort profile: Genomic data for 26 622 individuals from the Canadian Longitudinal Study on Aging (CLSA). *BMJ Open* **12**, (2022).
8. Kirkland, S. A. *et al.* Canadian Institutes of Health Research-Institute of Aging: Profile: Mining a Unique Canadian Resource: The Canadian Longitudinal Study on Aging. *Can. J. Aging* **34**, 366–377 (2015).
9. Tanigawa, Y. *et al.* Components of genetic associations across 2,138 phenotypes in the UK Biobank highlight adipocyte biology. *Nat. Commun.* **10**, (2019).
10. Watanabe, K., Taskesen, E., Van Bochoven, A. & Posthuma, D. Functional mapping and annotation of genetic associations with FUMA. *Nat. Commun.* **8**, 1–10 (2017).
11. Zhukovsky, P. *et al.* Multiscale neural signatures of major depressive, anxiety, and stress-related disorders. *Proc. Natl. Acad. Sci.* **119**, 1–10 (2022).
12. Morgan, S. E. *et al.* Cortical patterning of abnormal morphometric similarity in psychosis is associated with brain expression of schizophrenia-related genes. *Proc. Natl. Acad. Sci.* **116**, 9604–9609 (2019).
13. Makowski, C. *et al.* Larger cerebral cortex is genetically correlated with greater frontal. *Proc. Natl. Acad. Sci.* **120**, 1–10 (2023).
