# Supplementary material for: Genetic influences on brain and cognitive health and their interactions with cardiovascular conditions and depression
Source: Nat Commun. 2024 Jun 18;15:5207. doi: 10.1038/s41467-024-49430-7 (PMC11189393; doi:10.1038/s41467-024-49430-7)
Supplement: Supplementary file 3 — Description of Additional Supplementary Files [file 41467_2024_49430_MOESM3_ESM.pdf]

Description of Additional Supplementary Files

File Name: Supplementary Data 1
Description: Cohort descriptions

File Name: Supplementary Data 2
Description: Imaging Supplementary Data parameter description

File Name: Supplementary Data 3
Description: Meta-analytic GWAS results for the 13 associations between WMH and genomic risk loci

File Name: Supplementary Data 4
Description: Meta-analytic GWAS results for the 367 associations between CT phenotypes (corrected for eTIV) and genomic risk loci

File Name: Supplementary Data 5
Description: Clumping results across 34 cortical thickness GWAS

File Name: Supplementary Data 6
Description: Association statistics between SNPs identified in GWAS with memory and executive function (CLSA) and with Paired Associates Learning (UKB)

File Name: Supplementary Data 7
Description: Association statistics between polygenic scores (derived from global and regional CT GWAS) with memory and executive function in CLSA

File Name: Supplementary Data 8
Description: eQTL SNP-to-gene mapping of genomic risk loci for CT and comparison to TWAS results in ROSMAP

File Name: Supplementary Data 9
Description: Contingency tables for TWAS/GWAS results. Two-sided Fisher's exact tests were used

File Name: Supplementary Data 10
Description: Positional (ANNOVAR) SNP-to-gene mapping of genomic risk loci discovered in UKB

File Name: Supplementary Data 11
Description: Summary statistics for significant interactions between genomic risk loci for CT and cardiovascular health (GxE)

File Name: Supplementary Data 12
Description: Clumping results for significant interactions between genomic risk loci for CT and cardiovascular health (GxE)

File Name: Supplementary Data 13
Description: Summary statistics for significant interactions between genomic risk loci for CT and depression (GxE)

File Name: Supplementary Data 14
Description: Clumping results for significant interactions between genomic risk loci for CT and depression (GxE)

File Name: Supplementary Data 15
Description: Summary statistics for interactive effects of polygenic scores (dervied from global and regional CT GWAS) on cognitive function in CLSA

File Name: Supplementary Data 16
Description: Genetic correlations (LD score r_G) among regional CT calculated from the GWAS of regional measures corrected for eTIV

File Name: Supplementary Data 17
Description: Genetic correlations (LD score r_G) with selected phenotypes calculated from the GWAS of CT and WMH measures corrected for eTIV

File Name: Supplementary Data 18
Description: Genetic correlations - information on summary statistics

File Name: Supplementary Data 19
Description: Associations between MAPT haplotype tagging SNPs and cortical thickness

File Name: Supplementary Data 20
Description: Re-analysis of the GWAS results including a more expansive set of covariates

File Name: Supplementary Data 21
Description: Summary statistics for cross-validation of interactions between genomic risk loci for CT and cardiovascular health (GxE)

File Name: Supplementary Data 22
Description: Summary statistics for cross-validation of interactions between genomic risk loci for CT and depression (GxE)

File Name: Supplementary Data 23
Description: Anterior-posterior order of the risk loci for regional cortical thickness on Chromosome 17

File Name: Supplementary Data 24
Description: Summary statistics of the TWAS analysis of caudal middle frontal thickness in ROSMAP (extra file)

File Name: Supplementary Data 25
Description: Summary statistics of the TWAS analysis of rostral middle frontal thickness in ROSMAP (extra file)
