# Supplementary material for: Genetic influences on brain and cognitive health and their interactions with cardiovascular conditions and depression
Source: Nat Commun. 2024 Jun 18;15:5207. doi: 10.1038/s41467-024-49430-7 (PMC11189393; doi:10.1038/s41467-024-49430-7)
Supplement: Supplementary file 7 — Reporting Summary [file 41467_2024_49430_MOESM7_ESM.pdf]

nature portfolio

Corresponding author(s): Daniel Felsky

Last updated by author(s): Dec 7, 2023

Reporting Summary

Nature Portfolio wishes to improve the reproducibility of the work that we publish. This form provides structure for consistency and transparency in reporting. For further information on Nature Portfolio policies, see our Editorial Policies and the Editorial Policy Checklist.

Statistics

For all statistical analyses, confirm that the following items are present in the figure legend, table legend, main text, or Methods section.

n/a	Confirmed	
☐	☒	The exact sample size (*n*) for each experimental group/condition, given as a discrete number and unit of measurement
☐	☒	A statement on whether measurements were taken from distinct samples or whether the same sample was measured repeatedly
☐	☒	The statistical test(s) used AND whether they are one- or two-sided<br>*Only common tests should be described solely by name; describe more complex techniques in the Methods section.*
☐	☒	A description of all covariates tested
☐	☒	A description of any assumptions or corrections, such as tests of normality and adjustment for multiple comparisons
☐	☒	A full description of the statistical parameters including central tendency (e.g. means) or other basic estimates (e.g. regression coefficient) AND variation (e.g. standard deviation) or associated estimates of uncertainty (e.g. confidence intervals)
☐	☒	For null hypothesis testing, the test statistic (e.g. *F*, *t*, *r*) with confidence intervals, effect sizes, degrees of freedom and *P* value noted<br>*Give P values as exact values whenever suitable.*
☒	☐	For Bayesian analysis, information on the choice of priors and Markov chain Monte Carlo settings
☐	☒	For hierarchical and complex designs, identification of the appropriate level for tests and full reporting of outcomes
☐	☒	Estimates of effect sizes (e.g. Cohen's *d*, Pearson's *r*), indicating how they were calculated

Our web collection on statistics for biologists contains articles on many of the points above.

Software and code

Policy information about availability of computer code

Data collection	In this study, no data was collected as we analyzed data collected by the UK Biobank, ROS/MAP, and CLSA groups

Data analysis	MRI processing FreeSurfer<br>GWAS analyses: plink2<br>Post GWAS analyses: FUMA fuma.ctglab.nl/<br>Gene expression: limma (v3.48.3) voom in R (v4.1.1)<br>LDscore regression: LDSC package<br>LD r2 for pairs of SNPs: LDlink software in R;<br>LD clumping: plink1.9<br>Linear models: fitlm MATLAB R2016a<br>PLS regression: plsregress.m MATLAB R2016a<br>Modularity calculation from region-to-region genetic correlations: Brain Connectivity Toolbox, https://sites.google.com/site/bctnet/<br>Visualization of LD of selected SNPs on Chromosome 17: Haploreg v4.1 https://pubs.broadinstitute.org/mammals/haploreg/haploreg.php<br>Visualization of GO terms: clusterProfiler package in R (4.2.0)<br>Visualization of cell types: SEA-AD comparative viewer https://portal.brain-map.org/explore/seattle-alzheimers-disease<br>Visualization of brain maps: FreeSurfer Freeview (+ custom code to derive colormaps from brain map statistics)<br>All code published at https://github.com/peterzhukovsky/imaging_genetics DOI: 10.5281/zenodo.10895139 https://zenodo.org/records/10904367

For manuscripts utilizing custom algorithms or software that are central to the research but not yet described in published literature, software must be made available to editors and reviewers. We strongly encourage code deposition in a community repository (e.g. GitHub). See the Nature Portfolio guidelines for submitting code & software for further information.

Data

Policy information about availability of data

All manuscripts must include a data availability statement. This statement should provide the following information, where applicable:

- Accession codes, unique identifiers, or web links for publicly available datasets
- A description of any restrictions on data availability
- For clinical datasets or third party data, please ensure that the statement adheres to our policy

The GWAS summary statistics generated in this study have been deposited with the GWAS catalog (https://www.ebi.ac.uk/gwas/) under GCP ID: GCP000859. Raw data are available under restricted access as follows. Data are available from the Canadian Longitudinal Study on Aging (www.clsa-elcv.ca) for researchers who meet the criteria for access to de-identified CLSA data. Data are available from the UK Biobank (application #61530) for researchers who meet the criteria for access to de-identified UK Biobank data. The UK Biobank is a uniquely powerful biomedical database. It aims to facilitate research in life sciences by providing multiscale data for a large number of participants. The UK Biobank legally binds the researchers using the data not to publicly share UK Biobank data. Therefore, we are unable to share the data in a public repository. However, all data used here can be accessed by making a request with the UK Biobank. The UK Biobank has a dedicated portal for applying for data access here: https://www.ukbiobank.ac.uk/enable-your-research/apply-for-access. The use of UK Biobank data is not entirely free, but the data access costs are accessible to researchers. Researchers can submit a data request for ROS/MAP data to the Rush Alzheimer's Disease Center. SEA-AD data resource is publicly available at https://knowledge.brain-map.org/data as part of the Seattle Alzheimer’s Disease Brain Cell Atlas Comparative Viewer. The summary statistics for significant genetic variants are also available in the Supplementary Information.

Research involving human participants, their data, or biological material

Policy information about studies with human participants or human data. See also policy information about sex, gender (identity/presentation), and sexual orientation and race, ethnicity and racism.

Reporting on sex and gender	Individual-level data on sex was available. We conducted all analyses in men and women, while covarying for sex and age x sex interactions. We did not split our sample to conduct sex-specific analyses to ensure sufficient statistical power. It's costly to collect MRI and neurocognitive data alongside genetic information, which limits larger sample collection. In addition, testing for GxE interactions requires even more power to detect effects, which further discouraged us to split the sample for sex-stratified analyses. Future studies with larger sample sizes should conduct sex-stratified analyses to advance precision medicine.
Reporting on race, ethnicity, or other socially relevant groupings	Given the sample makeup of the UKB, ROS/MAP and CLSA, our participants were European. Future work in populations with diverse ancestries (e.g. AllOfUs) will be critical in identifying novel genetic mechanisms underlying risk and resilience for Alzheimer's Disease and related dementias.
Population characteristics	The UKB participants were between 45 and 81 years old (mean=63.8; SD=7.5). We did not exclude participants for the presence of specific medical conditions. Approximately 21% of the UKB participants self-reported having cardiovascular conditions and approximately 11% of the UKB participants self-reported having some depressive symptoms (PHQ-2).<br><br>ROS/MAP participants were between 55.4 and 90.5 years old (mean=75.9; SD=7.0). We did not exclude participants for the presence of specific medical conditions. Approximately 55% of the ROS/MAP participants self-reported having cardiovascular conditions. Presence of depressive symptoms was very low in the ROS/MAP sample.<br><br>CLSA participants were between 45 and 85 years old (mean=62.8 SD=10.2). We did not exclude participants for the presence of specific medical conditions. Approximately 37% of the CLSA participants self-reported having cardiovascular conditions. Presence of depressive symptoms was very low in the CLSA sample.<br><br>More details can be found in Supplementary Table 1.
Recruitment	UK biobank recruited participants from the community. According to Fry et al. (https://pubmed.ncbi.nlm.nih.gov/28641372/),

	compared with the general population, UK Biobank participants were more likely to be older, female, and to live in less socioeconomically deprived areas. UKB participants were less likely to be obese, to be a smoker, and to drink alcohol on a daily basis and had fewer self-reported health conditions.<br><br>CLSA also recruited participants from the community through the Canadian Community Health Survey (CCHS). The CCHS is conducted by Statistics Canada biannually aiming to provide cross-sectional measures of health determinants, health status, and health system utilization across Canadian provinces.<br><br>ROS enrolled nuns, priests, and brothers from across the US starting in 1994. MAP enrolled lay persons from across northeastern Illinois starting in 1997.
Ethics oversight	For UK Biobank data, ethical approval was obtained from from the National Health Service National Research Ethics Service (reference: 11/NW/0382) and all participants provided informed consent. UK Biobank also possesses a Human Tissue Authority (HTA) licence, so a separate HTA licence is not required by researchers who receive samples from the resource.<br>For ROS/MAP data, All ROS/MAP, participants provided informed and repository consent and also signed the Anatomical Gift Act, and ethical approval was obtained from the institutional review board of Rush University Medical Center.<br>For CLSA data, ethical approval was obtained from research ethics boards of all the participating institutions across Canada and informed consent was obtained from all participants.

Note that full information on the approval of the study protocol must also be provided in the manuscript.

Field-specific reporting

Please select the one below that is the best fit for your research. If you are not sure, read the appropriate sections before making your selection.

☒ Life sciences ☐ Behavioural & social sciences ☐ Ecological, evolutionary & environmental sciences

For a reference copy of the document with all sections, see nature.com/documents/nr-reporting-summary-flat.pdf

Life sciences study design

All studies must disclose on these points even when the disclosure is negative.

Sample size	Sample sizes were:<br>n=35,846 for UKB GWAS and GxE analyses (whole brain); n=22,791 for UKB analyses testing for associations between genotype and paired associates learning; n=134,640 for UKB analyses testing for associations between genotype and fluid intelligence<br>n=203 for ROS/MAP analyses testing for associations between genotype and cortical thickness (whole brain)<br>n=66 for ROS/MAP analyses testing for associations between differential gene expression and cortical thickness of the<br>n=25,387 for CLSA analyses testing for associations between genotype or PRS and memory/executive function<br><br>Sample sizes were determined by the available datasets featuring both neuroimaging and genetic data (UKB) or datasets featuring both cognitive and genetic data. Previous studies of neuroimaging phenotypes have used these datasets, showing that they have sufficient power to identify some main effects of genetic variation on these phenotypes. No a priori sample size calculations were ran.
Data exclusions	In MRI analyses, we excluded outliers (>4SD from the mean) in order to filter out failed FreeSurfer reconstruction data<br>We did not exclude participants with specific medical conditions<br>Inclusion criteria in UKB and ROS/MAP required presence of MRI and genotyping data<br>Inclusion criteria in CLSA required presence of cognitive and genotyping data
Replication	We followed up our UKB GWAS results with several analyses. First, we tested for associations of candidate variants from the UKB with cortical thickness in ROS/MAP. We found some variants to replicate in ROS/MAP; although many did not reach the suggestive threshold of p<0.05, this may be due to the differences in sample makeup (e.g. age, health conditions, religious population in ROS/MAP) and the small sample size in UKB. Next, we tested whether genes identified through the eQTL analyses of the UKB GWAS results were the same as those identified in ROS/MAP differential gene expression analyses. We identified several genes that were concordant across these two analyses, although a number of genes were specific to each analysis.<br><br>Further, we tested whether the genetic variants representing genetic risk loci for cortical thickness in UKB were also associated with cognitive function in a separate sample of older adults (CLSA). In CLSA, we found 'out-of-sample' main effects of polygenic scores on cognition. These polygenic scores were based on the UKB GWAS results for cortical thickness.<br><br>We adopted two approaches to validate the robustness of our GxE results. First, we used cross-validation in the UKB sample to test the robustness of our GxE results, finding that 27 of the 95 SNPs showing a GxE interaction in the whole sample also reached the replication threshold of p<0.05 in the cross-validation analysis. Second, extending our analyses of GxE effects on cortical thickness, we also found 'out-of-sample' interactive effects of polygenic scores (based on UKB results) with cardiovascular conditions in CLSA.
Randomization	NA - this was not a treatment study, but a cross-sectional association study
Blinding	NA - this was not a treatment study, but a cross-sectional association study

Reporting for specific materials, systems and methods

We require information from authors about some types of materials, experimental systems and methods used in many studies. Here, indicate whether each material, system or method listed is relevant to your study. If you are not sure if a list item applies to your research, read the appropriate section before selecting a response.

Materials & experimental systems

n/a	Involved in the study
☒	☐ Antibodies
☒	☐ Eukaryotic cell lines
☒	☐ Palaeontology and archaeology
☒	☐ Animals and other organisms
☒	☐ Clinical data
☒	☐ Dual use research of concern
☒	☐ Plants

Methods

n/a	Involved in the study
☒	☐ ChIP-seq
☒	☐ Flow cytometry
☐	☒ MRI-based neuroimaging

Plants

Seed stocks: NA

Novel plant genotypes: NA

Authentication: NA

Magnetic resonance imaging

Experimental design

Design type: Only structural MRI was used, no task or resting fMRI

Design specifications: NA

Behavioral performance measures: NA

Acquisition

Imaging type(s): structural

Field strength: 3T (UKB, ROS/MAP), 1.5T (ROS/MAP)

Sequence & imaging parameters: SIEMENS MAGNETOM Skyra syngo MR D13: MPRAGE 208 slices, TR = 2000ms TE = 2.01 ms, TI = 880 ms, FoV = 256 × 256 mm2, voxel resolution = 1 × 1 × 1 mm3, flip angle = 8 deg
UC 3T Philips Achieva: MPRAGE 181 slices, TR/TE (ms) = 8.1 / 3.7, TI = 961.89 ms, FoV = 240 × 228 mm2, voxel resolution = 1 × 1 × 1 mm3
Morton Grove 3T Siemens Trio: MPRAGE 176 slices, TR = 2300ms, TE=2.98ms, TI = 900 ms, FoV = 256 × 256 mm2, voxel resolution = 1 × 1 × 1 mm3
Bannockburn 1.5T GE Signa: SPGR 160 slices, TR/TE (ms) = 6300 / 2.8, TI = 1000 ms, FoV = 224 × 192 mm2, voxel resolution = 1 × 1 × 1 mm3, flip anle = 8 degrees
We also provide full details of the sequences in the Supplementary Table 2.

Area of acquisition: Whole brain

Diffusion MRI: ☐ Used ☒ Not used

Preprocessing

Preprocessing software: FreeSurfer
For UKB, image preprocessing was done by the UK Biobank team Freesurfer, with more details available at DOI: /10.1016/

	j.neuroimage.2017.10.034
Normalization	Cortical thickness values in the Desikan-Killiany parcellation and in the HCP (Glasser et al. 2016) parcellation were obtained in subject space; total intracranial volume measures (subject space) were also used as a covariate
Normalization template	Summary measures for each parcellaton and the TIV measures were obtained in subject space; for this purpose, FreeSurfer provides registration/normalization from subject space to the fsaverage space
Noise and artifact removal	No fMRI data used; excluded Outliers (4 SD from the mean)
Volume censoring	NA (no fMRI data used)

Statistical modeling & inference

Model type and settings	UKB: General linear models (plink2, MATLAB fitlm)<br>ROS/MAP: mixed effect linear model fitting a random intercept for each subject given that many participants had multiple MRI datapoints (fitlme, MATLAB)
Effect(s) tested	GWAS: effect of genotype (effect allele) on cortical thickness (global and 33 bilateral regions) and on white matter hyperintensities (UKB)<br>Effect of genotype (220 candidate SNPs from genomic risk loci from GWAS) on cortical thickness (ROS/MAP)<br>Effect of genotype (220 candidate SNPs from genomic risk loci from GWAS) on executive function (CLSA), memory (CLSA), paired associates learning (UKB) and fluid intelligence (UKB)<br>Effect of polygenic scores on executive function (CLSA) and memory (CLSA)<br>Effect of cortical thickness of rostral and caudal middle frontal gyri on differential gene expression in ROSMAP<br>GxE: Interaction of genotype (220 candidate SNPs from genomic risk loci from GWAS) x cardiovascular conditions on regional cortical thickness (UKB)<br>GxE: Interaction of genotype (220 candidate SNPs from genomic risk loci from GWAS) x depressive symptoms on regional cortical thickness (UKB)<br>GxE: Interaction of polygenic scores x cardiovascular conditions interactions on executive function and memory (CLSA)

Specify type of analysis: ☐ Whole brain ☐ ROI-based ☒ Both

Anatomical location(s)	We conducted differential gene expression analyses in the caudal middle frontal and rostral middle frontal gyri in ROS/MAP data since these regions approximately cover the dorsolateral prefrontal cortex, from which post mortem tissue was taken<br>All other analyses were conducted in the whole brain

Statistic type for inference<br>(See Eklund et al. 2016)	NA
Correction	GWAS analyses: GWAS p-threshold of 5e-8; also reporting the more stringent threshold of 5e-8/34 for 34 cortical thickness GWAS<br>Region-specific polygenic scores: FDR correction across regions, FDR<0.1<br>We also tested for the effects of 220 unique genetic variants representing genomic risk loci identified in UKB GWAS on cognition in CLSA: FDR correction, FDR<0.1<br>Case-control comparisons of regional cortical thickness (n=360 ROIs in the HCP atlas): FDR<0.05<br>Differential gene expression analyses in ROS/MAP: FDR<0.05

Models & analysis

n/a	Involved in the study
☒	☐ Functional and/or effective connectivity
☒	☐ Graph analysis
☐	☒ Multivariate modeling or predictive analysis

Multivariate modeling and predictive analysis	Supplementary analysis: partial least squares regression; permutation testing for significance
